# Diverse soil protists show auxin regulated growth in partnership with auxin-producing bacteria

**DOI:** 10.1093/ismejo/wraf234

**Published:** 2025-10-16

**Authors:** Ravikumar R Patel, Lindsay R Triplett, Stephen J Taerum, Sara L Nason, Cole O Wilson, Blaire Steven

**Affiliations:** Department of Plant Pathology and Ecology, the Connecticut Agricultural Experiment Station, New Haven, CT 06511, United States; Department of Plant Pathology and Ecology, the Connecticut Agricultural Experiment Station, New Haven, CT 06511, United States; Department of Plant Pathology and Ecology, the Connecticut Agricultural Experiment Station, New Haven, CT 06511, United States; Department of Environmental Science and Forestry, the Connecticut Agricultural Experiment Station, New Haven, CT 06511, United States; Department of Plant Pathology and Ecology, the Connecticut Agricultural Experiment Station, New Haven, CT 06511, United States; Department of Environmental Science and Forestry, the Connecticut Agricultural Experiment Station, New Haven, CT 06511, United States

**Keywords:** protist, indole-3-acetic acid (IAA), auxin, plant beneficial bacteria, plant-growth promoting traits, *Colpoda*, metagenome, interkingdom signaling

## Abstract

Predatory protists are single-cell eukaryotic organisms capable of hunting and ingesting bacteria and other microorganisms, which are thought to enrich populations of beneficial bacteria in the rhizosphere, potentially influencing plant health. However, the mechanisms underpinning protist interactions with plant growth promoting bacteria are not well understood. We examined the conservation of plant beneficial traits in bacteria associated with 10 protists of diverse lineages that were isolated from the maize rhizosphere. Metagenomics, whole-genome sequence analysis, and functional assays of 61 groups of protist-associated bacteria identified tryptophan-dependent biosynthesis of the auxin hormone indole-3-acetic acid (IAA) as the most prevalent predicted trait. Mass spectrometry confirmed that all the protist cultures accumulated IAA after tryptophan supplementation, and that IAA production was bacterial-dependent. Hypothesizing that IAA affects protist function, we observed that exogenous IAA significantly increased the culture density and cell size of all 10 protists. Examination of four partial protist genome assemblies identified 13 candidate auxin metabolic gene homologs conserved across plants and protists, and transcriptomic analysis of a *Colpoda* sp. protist revealed differential expression of thousands of genes in the presence of IAA, further supporting auxin regulation of protist function. These findings demonstrate that soil microeukaryotes can widely host auxin-producing bacteria and that much broader range of eukaryotic lineages perceive and respond to auxin signals than previously recognized. This significantly expands the known breadth of auxin perception as an interkingdom signal, with important implications for soil nutrient cycling and rhizosphere ecology.

## Introduction

Heterotrophic protists are diverse eukaryotes that are integral to soil and aquatic microbial food webs. Selective predation by protists facilitates the transfer of organic carbon and nutrients to higher trophic levels, increasing the fitness of predation-resistant bacteria [1, 2]. Beyond predator–prey interactions, protists may also shape bacterial communities through parasitic, mutualist, or symbiotic associations [3, 4]. Protists interact with a remarkable functional diversity of internal (endosymbiotic) and external (ectosymbiotic) bacteria in a transient or stable manner, exchanging nutrients, energy, providing detoxification, and other services [4]. Bacteria from the genus *Mesorhizobium* produce vitamin B_12_ for *Chlamydomonas reinhardtii* [5], *Marinobacter* perform iron acquisition for dinoflagellates and coccolithophores [6], *Candidatus azoamicus* produces energy for its ciliate host [7], and several bacteria perform nitrogen fixation for protists in the termite gut [8]. In addition to nutrient and energy exchange, signaling molecules such as tryptophan, bacterial-excreted ammonium, diatom-excreted organosulfur, siderophores, quorum-sensing compounds, and volatile organic compounds mediate communications between protists and bacteria, influencing behaviors like symbiotic associations, bacterial aggregation, and chemotaxis [9–11].

In the rhizosphere, defined as the zone of soil influenced by plant roots, protists affect plant growth, and rhizosphere ecology by mineralizing microbial nitrogen and carbon, culling inactive bacteria, and reducing pathogen load [2, 12–15]. Protists may also promote fitness and root colonization of certain plant growth promoting rhizobacteria (PGPR) [12, 16–18]. Predation favors activation of plant growth promoting (PGP) traits that confer predation resistance, such as antibiosis, biofilm formation, and motility [14, 19], and internalization by protists facilitates bacterial distribution along the root and promotes long-term persistence in soil [17, 20]. We previously observed that many species of root-colonizing bacteria persist in protist cultures, and that the bacteria protect plants from growth-suppressing soil microbes [21, 22] Yet, the mechanisms and roles of PGP traits in mediating bacteria-protist interactions are largely unknown.

We hypothesized that bacterial traits associated with beneficial plant outcomes may also be involved in shaping protist interactions. To test this, we analyzed PGP genes that were conserved among the bacterial communities associated with diverse protist isolates from the maize rhizosphere. Metagenomics and functional assays determined that production of the master regulatory phytohormone indole-3-acetic acid (IAA) was the most prevalent trait surveyed, and tryptophan supplementation resulted in bacterial-dependent IAA accumulation in protist cultures. All protists responded to exogenous IAA, which increased protist culture density and cell size. Thousands of *Colpoda* sp. transcripts were differentially abundant in response to IAA, including homologs to plant IAA response genes. The findings demonstrate that diverse lineages of eukaryotes perceive and respond to IAA, implicating the hormone as a broad-spectrum interkingdom signal in the rhizosphere.

## Materials and methods

### Protist cultures

The protists employed in this study were isolated from maize rhizosphere samples collected from Lockwood and Griswold farms in Connecticut, USA [23]. In brief, root tissue was incubated in sterile water for 2 days, and the resulting suspension was diluted until 1 μL droplets contained single protist cells. These were transferred into 96-well plates with PAGE’s saline solution, soil extract, and heat-killed *Escherichia coli* as a food source, and subsequently propagated into uniform cultures. After isolation, all these protists were maintained in PAGE’s amoebal saline, amended with 5.6 × 10^6^ cells ml^−1^ of heat-kill *E. coli* at 22°C temperature in the dark as described [21]. At the start of this study, the cultures had been transferred to fresh media monthly 18 times without plant contact. Bacterial-depleted *Colpoda* sp. were generated using the method of repeated multiple antibiotic treatment described in [21], with an additional modification: *Colpoda* sp. was treated with streptomycin (50 μg ml^−1^) for five days, followed by a second identical treatment for another five days. After antibiotic treatment, the protists were transferred back into PAGE’s containing heat-killed *E. coli*. To verify and characterize protist morphology, all 10 protist species were assessed by comparative microscopy (Supplementary Note: Method S1).

### Protist culture DNA extraction

Each 2 ml protist culture was inoculated into two sterile 50 ml Nunc polypropylene tubes containing 18 ml fresh PAGE’s with 5.6 × 10^7^ heat-killed *E. coli* ml^−1^. Cultures were incubated in the dark at 22°C for five days, then vortexed and centrifuged at 3000 × g for 20 min. The supernatant was discarded, and the pellet was resuspended in the residual medium and centrifuged again under the same conditions. The cells were suspended in 2 ml PAGE’s, vortexed and divided into two tubes: one used for DNA isolation with a ZymoBIOMICS DNA Miniprep Kit (Zymo Research, Irvine, CA, USA), and the other for the cultivation of protist associated bacteria.

### 18S rRNA gene amplification and sequencing

To validate the protist cultures after serial passaging, 18S rRNA genes were amplified using genomic DNA extracted previously [24–26] and sequenced by Sanger sequencing at the Yale School of Medicine (New Haven, CT, USA). A detailed PCR protocol is provided in Supplementary Note: Method S2.

### Shotgun metagenomic sequencing and assembly

Metagenomic libraries were prepared from the extracted DNA using the Illumina DNA Prep Kit (Illumina, San Diego, CA) and adjusted to 180 ng in a total of 30 μL. Nextera DNA CD indexes were added to individual libraries for sequence multiplexing. Sequencing was performed at the Yale Center for Genome Analysis, Yale University (New Haven, CT) on the NovaSeq 6000 System (Illumina) platform, with a targeted sequencing depth of 10 million reads per sample, using 2x150 sequencing chemistry.

Raw sequencing data in FASTQ format were imported into KBase [27]. Bioinformatic pipelines are available in the KBase narrative “Metagenome_10protists (Metgenome_10protists—KBase Narrative). Briefly, paired ends were trimmed using Trimmomatic v0.36 with the NexteraPE-PE adapter clipping option [28]. Contigs were assembled using metaSPAdes v3.13.0 with a minimum length of 300 bp [29]. Assemblies were performed on each protist culture separately as well as on a merged sample of all reads. The resulting contigs were downloaded from KBase to screen for eukaryotic sequences using Tiara v1.0.1 [30].

To obtain bacterial MAGs from the assembled contigs, we used three complementary binning tools via KBase applications: Concoct v1.1 [31], MaxBin2 v2.2.4 [32], and MetaBAT2 v1.7 [33], which each uses different algorithms for binning. The resulting bins were then refined to generate consensus MAGs using DAS-Tool v1.1.2 [34], which integrates and dereplicates bins from multiple sources to generate a nonredundant, high-quality set of MAGs. MAG quality was assessed using CheckM v1.0.18 [35]. Finally, the assembled bacterial MAGs were taxonomically classified using the GTDB-Tk [36] in KBase.

Eukaryotic metagenomic contigs were uploaded to KBase in a separate narrative named Euks_binning, where eukaryotic contigs were binned using Concoct v1.1 [31]. In addition to short-read sequencing, we performed long-read sequencing of *Colpoda* sp. and generated a hybrid genome assembly using both short and long reads (Supplementary Note: Method S3). The completeness of protist genomes was assessed using BUSCO v5 ortholog Eukaryota of gVolante [37]. The four protist genomes with more than 50% completeness were selected for analysis of auxin-related genes. Structural gene prediction was performed on all four genomes using Augustus (v4.0.0) (with *Tetrahymena thermophila* as the reference and “complete genes only” enabled) [38]. These genomes were queried using tBLASTn with the 44 auxin-related protein-coding genes from *Arabidopsis thaliana* [39, 40]. Searches were run with default parameters (including a word size of 11) and filtered to retain hits with ≥30% translated nucleotide identity, ≥85% query coverage, and an E-value ≤1e–7. For each gene, the top hit was selected based on the lowest E-value and highest identity.

### Isolation and typing of bacteria

To isolate protist-associated bacteria, washed and concentrated protist samples were homogenized by vortexing and serially diluted in 0.8% NaCl solution. 100 μL of the 1:100 000, 1:10 000, and 1:1000 dilutions were then spread onto Reasoner’s 2A (R2A) agar plates [41] and incubated at 28°C for 96 h. Colonies representing distinct morphologies were selected, restreaked onto R2A plates for purification, and cryopreserved from the plates in 15% glycerol at −80°C. For DNA isolation, frozen stocks were first revived on plates, followed by growth in liquid culture to obtain sufficient biomass for DNA extraction using the ZymoBIOMICS DNA Miniprep Kit (Zymo Research, Irvine, CA, USA). The 16S rRNA genes from 115 isolates were amplified (Supplementary Note: Method S2) [42, 43] and sequenced at the Yale School of Medicine, Yale University, New Haven, CT, USA. Bacteria were enumerated from *Colpoda* sp. by dilution plating on R2A, using methods described in Supplementary Note: Method S4.

### Bacterial genome sequencing and assembly

DNA from 36 bacterial isolates was quantified using the Qubit V4 Fluorometer (Thermo Fischer Scientific, Waltham, MA, USA). Sequencing libraries were prepared using the Native Barcoding Kit 24 V14 (SQK-NBD114.24, Oxford Nanopore Technologies). DNA was sequenced in two MinION flow cells (FLO-MIN14, R10 Version, SN-B024048252) on the Oxford Nanopore MinION MK1C. Following sequencing, base calling was performed with the Dorado (v0.74) software using the super accurate “SUP” model. Quality control of raw reads was performed on the raw sequencing data using LongQC [44]. Genome assembly was performed using Flye, which is a long-read de novo genome assembly pipeline [45]. Polishing was performed using the Burrows-Wheeler Aligner (BWA) v0.7.17 [46]. and Racon v1.4.19 [47] with parameters specific for Nanopore read alignment and mapping. The polished output was further processed with Medaka v1.0.3 (https://github.com/nanoporetech/medaka).

### Bacterial genome analysis

Pairwise genome average nucleotide identity (ANI) analyses were conducted in the KBase platform [27, 48] (“ANI_MAGs” ANI_MAGs—KBase Narrative). A threshold of 95% ANI was utilized to assign MAGs and isolate sequences to 61 genome groups. Protein-coding sequences were predicted in each genome group using MetaGeneMark [49], then annotated using the Plant Growth-Promoting Traits Prediction tool (PGPT-Pred) from PLabAse [50]. To identify 1-Aminocyclopropane-1-carboxylate (ACC) deaminase and secretion system genes, bacterial genomes were annotated in BlastKOALA of KEGG by selecting prokaryotes as reference gene dataset [51]. Qualitative assays for the production of siderophores, cellulase, chitinase, protease, indole, and phosphate solubilization was performed as previously described [52, 53].

### Indole-3-acetic acid detection

Each of the 32 bacterial isolates (200 μL) was grown in quadruplicate in 96-well plates at 28°C for 72 h, at which point most cultures were in the mid-stationary phase in half-strength R2A broth containing 100 μg ml^−1^ tryptophan. Detection of auxin was performed in quadruplicate using a modified Salkowski assay, a semiquantitative colorimetric assay that also detects some other indolic compounds [54]. A standard curve was established using synthetic IAA (GOLDBIO, St. Louis, MO, USA) in concentrations ranging from 10 to 50 μg ml^−1^.

IAA was quantified in protist cultures using LC-HRMS. Protists were cultured in a total of 20 ml PAGEs started with an initial inoculum of 1000 cells ml^−1^ for each protist and grown at 22°C in the dark for 72 h, with supplementation of heat-killed *E. coli* (5.6 × 10^7^ cells ml^−1^) and 100 μg ml^−1^ of tryptophan. Triplicate cultures were filtered through 0.22 μm sterile filters (MilliporeSigma) to remove cells, and auxin was extracted by three consecutive liquid–liquid extractions with ethyl acetate [55]. Extracts were filtered through a 0.45 μm nylon 13 mm mini disposable membrane filter prior to LC-HRMS analysis. IAA quantification was performed using methods previously described [56] (Supplementary Note: Method S5).

### Assay for protist response to indole-3-acetic acid and analogs

To evaluate the effects of IAA supplementation on the protist growth, protists were grown for 96 h at 22°C in the dark then inoculated at 1000 cells ml^−1^ into 2 ml of PAGE’s medium supplemented with IAA or solvent control (0, 0.001, 0.01, 0.1, 1, 10, 100, or 200 μM IAA in 2,3 methanol,water) and heat-killed *E. coli* (5.6 × 10^7^ cells ml^−1^). Assays were performed in sterile 24-well flat-bottom cell culture polystyrene plates (Corning Inc., Corning, NY, USA); six biological replicate wells were imaged daily for five days using a Zeiss ID02 Invertoscope inverted microscope. Three fields of vision per well were photographed at 200× with an Axiocam 305 color camera (*Colpoda* and *Allapsa* spp. were imaged at 100×). Cells were manually counted using the Cell Counting plugin in ImageJ v1.54g and adjusted to cells ml^−1^. Counts were analyzed by fitting a logistic growth curve model using the base R (version 4.5.0) functions, accessed via RStudio v2024.12.0. Doubling time for each protist under each treatment condition was calculated from the exponential phase of the fitted model by determining the time required for the population to double in size. The same enumeration protocol was used to measure protist density 72 h after treatment with 2,4-dichlorophenoxyacetic acid (2,4-D, Sigma-Aldrich Co., St. Louis, MO, USA) or glucose (Fisher Chemical, Fair Lawn, NJ, USA), and *Colpoda* density with and without supplementation of heat-killed *E. coli*.

Protist cell area was measured using ImageJ v1.54g by first setting the scale according to the image’s scale bar, followed by manual outlining of individual cells using the Freehand Selection tool to obtain area measurements. A total of 100 randomly selected active cells were measured from both control and IAA-treated cultures for each of the seven protist species. Measurements were taken at time points when most cells were active.

### Transcriptome sequencing and analysis

We performed transcriptome analysis of *Colpoda* sp. with and without 100 μM IAA treatment. After 72 h of growth, 2 ml of a *Colpoda* sp. culture was inoculated into four replicate tubes with 18 ml PAGE’s containing 5.6 × 10^7^ heat-killed *E. coli* ml^−1^, with or without IAA. After 72 h, cells were collected via 0.8 μm membrane filtration (Millipore Sigma, Burlington, MA, USA) and RNA was extracted from the filtered cells using the quick-RNA MiniPrep kit (Zymo Research, Irvine, CA), which includes an on-column DNase digestion step to remove genomic DNA. The RNA-seq library was prepared using the Zymo-Seq Ribo-Free Total RNA Library Kit (Zymo Research, Irvine, CA). A normalized 113 ng of RNA was used as input for the library preparation from each sample. After measuring the quality and concentration of each prepared library, they were pooled in equimolar concentrations and sequenced on the NovaSeq 6000 System (Illumina) at the Yale Genome Center, Yale University (New Haven, CT). We conducted a transcriptome analysis within KBase [27]. Reads were trimmed using Trimmomatic v0.36 [28] and aligned to the reference genome with TopHat v1.0.1 [57]. Transcripts were assembled using StringTie v2.1.5 [58] and differential expression analysis was performed using DESeq2 v1.20.0 [59]. Functional annotations of differentially expressed genes were assigned using KEGG BlastKOALA (Eukaryote reference) [51]. Expression patterns of 32 auxin-related genes previously identified in the *Colpoda* genome were also evaluated.

### Statistical methods

All statistical analyses were conducted using R software 4.5.0 (accessed via RStudio v2024.12.0) and Microsoft Excel (Version 2503 Build 16.0.18623.20178). Comparisons of two groups were performed using two-tailed Student’s t-tests assuming equal variance. Comparisons among more than two groups were performed using one-way analysis of variance (ANOVA) followed by Tukey’s HSD post hoc test in R function (aov) and (TukeyHSD) (α = 0.05).

## Results and discussion

### Protist cultures host simple and novel bacterial communities

**Figure 1:**
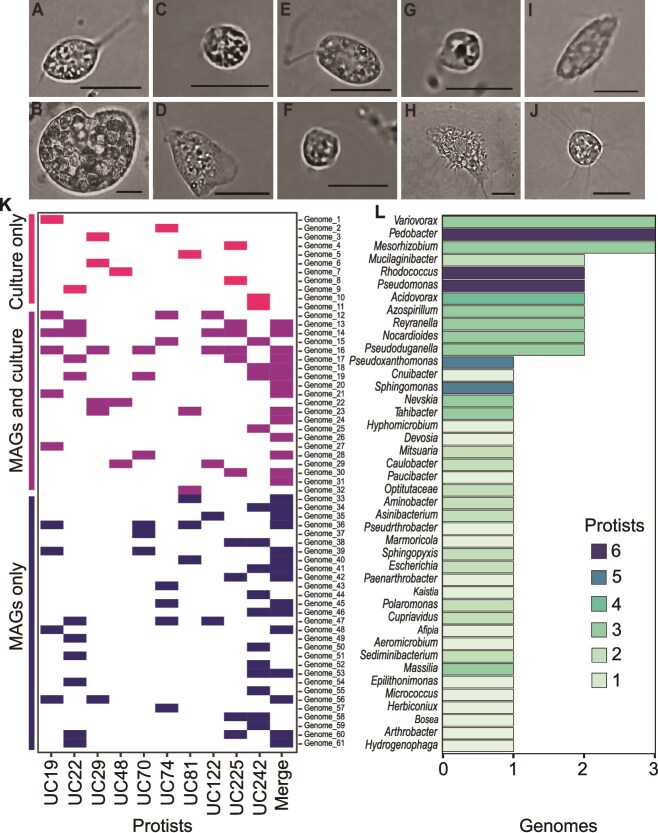
Morphology and taxonomic classification of protists and their associated bacteria. (A) UC19 (*Cercomonas* sp.); (B) UC22 (*Colpoda* sp.); (C) UC29 *(Ochromonas* sp. 1); (D) UC48 (*Poteriospumella* sp.); (E) UC70 (*Thaumatomastix* sp.); (F) UC74 (*Chrysophyte* sp.); (G) UC81 (*Ochromonas* sp. 2); (H) UC122 (*Flamella* sp.); (I) UC225 (*Allapsa* sp.); (J) UC242 (*Nuclearia* sp.). Images were taken under inverted light microscopy using an oil immersion lens (100X objective). The proposed designations for the 10 protists is based on 18S rRNA gene sequencing. The scale bar in each panel = 10 μm. A full description of the taxonomic classification for each strain is presented in extended data table 1. (K) Presence-absence heatmap for the 61 unique bacterial genomes denoting their conservation amongst the 10 protist cultures. Genomes were considered to belong to the same species if they exhibited an ANI ≥95%. The bars at the left of the graph denote the source of the genome sequences: MAGs only (from metagenomes), MAGs and cultured isolates (identified in both), and cultured isolates only. Each column represents a protist culture (table S1), whereas the column “merge” is the result of a combined metagenomic assembly across all reads from each protist culture. The full list of bacterial genomes and their taxonomic identification is presented in Data S1. (L) Taxonomic classification of genomes. Of the 61 genomes, 57 could be classified to the genus level, and 42 distinct genera were identified. The number of genomes classified to each genus is indicated by the bars. The number of protist cultures in which each genus was detected is represented by the bar color.

Ten protist cultures were selected from a collection of maize rhizosphere protists that had been maintained for 18 months in laboratory culture conditions [22] to represent morphological and taxonomic diversity, representing six phyla and nine families (Figs 1A–J). Metagenomic sequencing from 10 protist cultures produced 101 metagenome-assembled bacterial genomes (MAGs). Pairwise ANI identified unique MAGs (MAGs sharing ≥95% ANI were considered different genomes), producing a set of 50 distinct genome groups (Figs. S1 and S2).

Bacterial isolation from the cultures generated a library of 115 bacterial isolates. 16S rRNA gene sequencing placed the isolates in 32 distinct taxa (Fig. S3). Whole-genome sequencing was performed on representative isolates from each of the 32 unique taxa, with additional isolates included for the highly prevalent genera *Variovorax*, *Pseudomonas*, *Pseudoxanthomonas*, and *Pedobacter*, resulting in a total of 36 bacterial genomes. ANI analysis against the MAGs, showed that 23 isolate genomes (46%) were also represented by MAGs (≥ 95% ANI), indicating substantial recovery of bacterial diversity in culture and strong concordance of isolation-dependent and independent methods. Genomes of 11 isolates were not represented in the MAGs. Together, genomic and metagenomic methods produced assemblies of 61 unique bacterial genomes (Fig. 1K) (Data S1). Three to 16 genomes were recovered from each protist culture (median = 6.5).

Taxonomic classification of the genome sequences determined that 50 (82%) of the protist-associated genomes represented undescribed species, including three from undescribed genera and one from an undescribed family (Fig. S4). The majority (73%) of the 42 genera identified were represented by one genome (Fig. 1L). The genera with the most species diversity were *Variovorax*, *Pedobacter,* and *Mesorhizobium,* represented by three ANI groups each. *Pedobacter*, *Rhodococcus*, and *Pseudomonas* were the most prevalent genera and detected in six of the 10 cultures, underscoring that each protist culture was associated with a unique bacterial community. Because both metagenomic and culture datasets are biased toward abundant taxa, assembly datasets are likely to miss some rare species found in amplicon-based studies [22]. Nevertheless, the findings show that the bacterial populations of rhizosphere protist cultures are comprised of relatively simple bacterial communities, where the majority of taxa are novel and associated with a single protist isolate.

### Plant growth-promoting genes are common in protist-associated bacterial genomes

**Figure 2:**
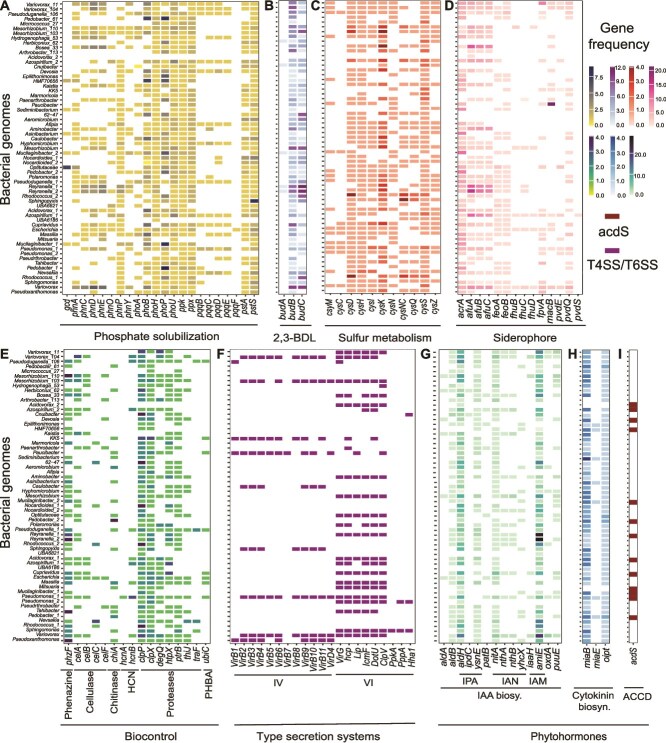
Distribution of plant growth-promoting and interaction-related genes in protist-associated bacterial genomes. (A–E) Presence of genes related to PGP functions: (A) phosphate solubilization, (B) 2,3-butanediol biosynthesis (2,3-BDL), (C) Sulfur metabolism, (D) siderophore biosynthesis and export, and (E) biocontrol activity, including genes encoding chitinase, cellulase, protease, phenazine, hydrogen cyanide (HCN), and 4-hydroxybenzoic acid (PHBA). (F–I) Prevalence of genes associated with interkingdom interactions and signaling: (F) type IV and type VI secretion systems, (G) IAA biosynthesis genes across major pathways (IAN, IAM, IPA), (H) cytokinin biosynthesis, and (I) ACC deaminase involved in ethylene modulation. Each row represents a bacterial genome, and each column corresponds to a gene associated with a specific functional trait. Color intensity reflects the gene copy number per genome; white boxes indicate gene absence. For secretion systems (F) and ACC deaminase (I), only binary presence/absence is shown. A full list of genome IDs and their taxonomic assignments is available in Supplementary Data S1.

To test the hypothesis that plant growth-promoting (PGP) traits are prevalent among protist-associated bacteria, we screened 61 unique representative bacterial genomes identified through metagenome sequencing and culture-based approaches for 93 key genes linked to well-characterized PGP functions. Several trends emerged across the dataset, revealing a consistent enrichment of traits that may support not only plant health but also influence interactions with protist hosts. Among nutrient-mobilization traits, genes associated with phosphate solubilization were widespread, particularly *pqqC/D*, *gcd*, and *phoA*, which were present in 28%, 10%, and 36% of genomes, respectively (Fig. 2A). These genes enable bacteria to convert insoluble soil phosphorus into bioavailable forms and are considered central to rhizosphere competence. Additionally, genes for the production of 2,3-butanediol, a volatile compound known to promote plant growth and induce systemic resistance, were detected in 77% of genomes (Fig. 2B) [60]. Siderophore biosynthesis genes, including *pvdEQS* and *macB*, were present in 20% of genomes (Fig. 2D), suggesting a potential role in iron acquisition and microbial fitness under nutrient-limited or predator-rich conditions [61, 62]. Out of the 10 protists examined, seven, *Cercomonas* sp., *Colpoda* sp., *Poteriospumella* sp., *Thaumatomastix* sp., *Chrysophyte* sp., *Flamella* sp., and *Nuclearia* sp. harbored at least one bacterial associate possessing all three key traits: phosphate solubilization, siderophore production, and 2,3-butanediol biosynthesis. This pattern suggests a functional redundancy among protist-associated bacteria toward traits that could benefit both plant hosts and their protist vectors, enhancing bacterial persistence and ecological flexibility. Additionally, genes involved in sulfur metabolism (65%) (Fig. 2C) and the biosynthesis of biocontrol compounds (19%) (Fig. 2E) further support the notion that protist-associated bacteria harbor multifunctional capacities that extend beyond basic nutrient acquisition to roles in inter-organismal defense and signaling. A qualitative analysis of several PGP traits demonstrated that for each protist examined, at least one co-isolated bacterium was positive for each of the following activities: siderophore production, indole, cellulase, chitinase, protease, and phosphate solubilization (Fig. S5). To further explore traits mediating direct protist–bacteria interactions, we screened for bacterial secretion systems. The type II secretion system was most common (34%), although type I, type IV, and type VI were present in 13%, 15%, and 33% of genomes, respectively. The type III secretion system was the least frequent (8%) (Data S1). Particularly, the co-occurrence of type IV and type VI secretion systems, which are typically involved in host interaction and microbial competition [62], was found in bacteria associated with five protist species: *Cercomonas* sp., *Poteriospumella* sp., *Thaumatomastix* sp., *Chrysophyte* sp., and *Flamella* sp. (Fig. 2F). Co-localization of T4SS and T6SS in a single genome may enable both intimate host interaction and defense against protist predation, suggesting a dual strategy for persistence within complex microbial environments.

Among all traits, phytohormone production was the most prevalent (Figs 2G–I). The cytokinin biosynthesis genes *ipt* and *maiB* were nearly ubiquitous, found in 98% and 95% of genomes, respectively (Fig. 2H). Genes associated with tryptophan-dependent IAA biosynthesis were present in 90% of genomes (Fig. 2G), spanning three canonical pathways: the indole-3-acetonitrile (IAN), indole-3-acetamide (IAM), and indole-3-pyruvate (IPA) routes, along with *aldA/B* dehydrogenase [63, 64]. Many genomes encoded components of all three IAA synthesis pathways, which match previous observations that single organisms frequently possess redundant, interconnected pathways for IAA biosynthesis. This redundancy provides resilience; if one pathway is disrupted, IAA production can still proceed [63]. All 10 protist species had at least two bacterial associates carrying core IAA biosynthesis genes, underscoring the central role of IAA as a likely mediator in protist–bacteria associations. Finally, ACC deaminase activity, required for ethylene disruption, was detected in 21% of bacteria (Fig. 2I). Together, these findings indicate that protist-associated bacteria possess a broad suite of functional traits traditionally associated with plant growth promotion. These adaptations could enhance bacterial survival, dispersal, and ecological versatility in multitrophic interactions.

### Validation of indole-3-acetic acid production

**Figure 3:**
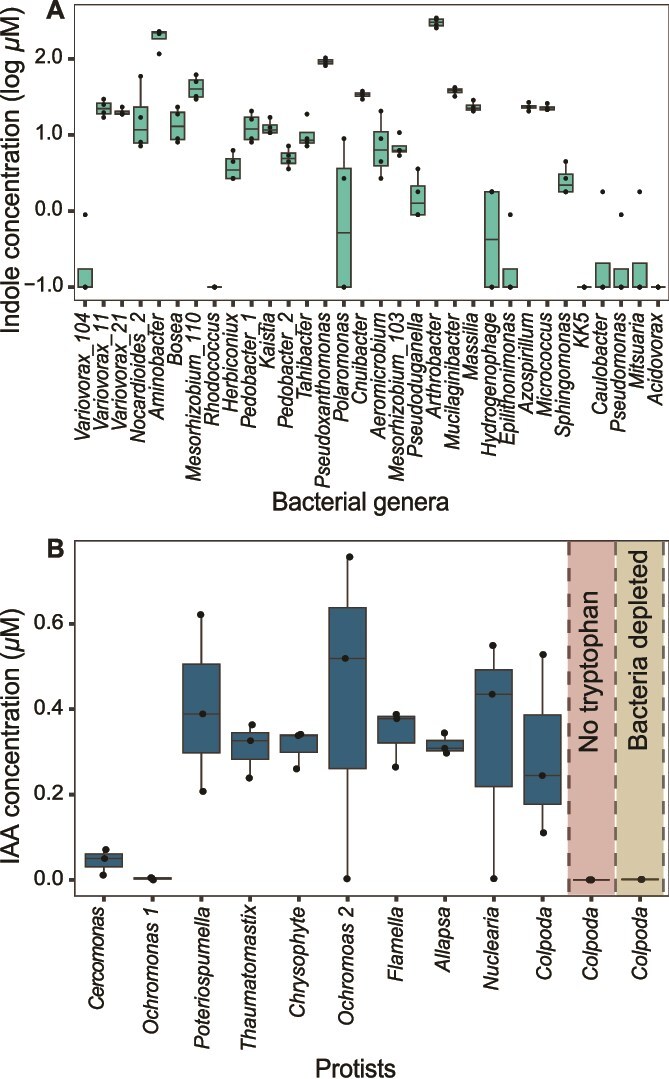
(A) Indole concentration in 32 distinct bacterial isolates after 100 μg ml^−1^ supplementation of tryptophan and after 72 h of growth. This experiment was performed with four biological replicates for each of the bacterial isolates. (B) IAA concentrations were determined by LC-HRMS in protist cultures after 72 h of growth in the presence of 100 μg ml^−1^ of tryptophan with three biological replicates. The no-tryptophan column shows IAA concentrations in the *Colpoda* sp. culture when tryptophan was omitted. The bacteria depleted column shows IAA concentration when bacterial diversity was reduced by antibiotic treatment (see methods). In both box graphs, the center line indicates the median, the box represents the interquartile range (IQR), and the whiskers represent 1.5 × IQR.

To validate the genomic predictions and conservation of IAA production by measured phenotypes, a colorimetric assay determined that 72% of bacterial isolates produced auxin in a manner dependent on tryptophan (Fig. 3A). To determine if bacteria maintained this activity in association with protists we performed liquid chromatography-high resolution mass spectrometry (LC-HRMS) on the protist cultures. All protist cultures accumulated IAA in the presence of tryptophan at concentrations ranging from 0.004 μM to 0.4 μM. IAA was not detected in a *Colpoda* sp., protist culture without supplemented tryptophan, supporting tryptophan dependence of IAA production (Fig. 3B). In order to confirm the role of bacteria in the production of IAA, bacteria were depleted from the *Colpoda* sp. culture, which abolished detection of IAA, supporting that bacteria are the main source of IAA in protist cultures (Fig. 3B). Collectively, these findings validate the genomic predictions of tryptophan-dependent IAA production within the bacterial communities associated with rhizosphere protists.

### Exogenous indole-3-acetic acid increases population and cell size of diverse protists

**Figure 4:**
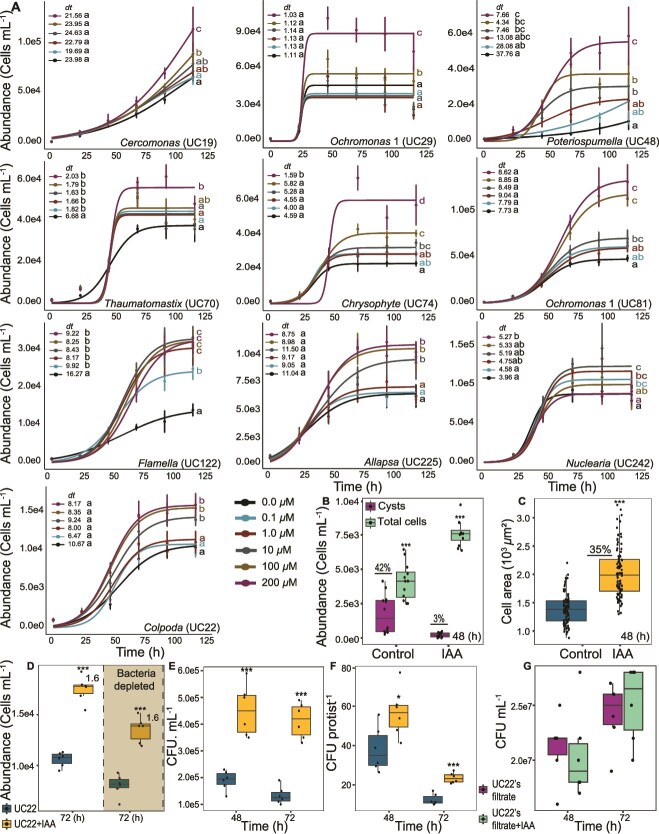
Growth response of protists to IAA. (A) Dose–response growth curves. Curves were generated after fitting the data into a logistic growth curve model; points represent median values with standard deviation error bars. Each curve is the result of six replicates. Lines labeled with different letters indicate statistically significant differences in final cell counts according to Tukey’s HSD post hoc test (α = 0.05). The error bar represents the standard deviation across replicates. Average doubling time was calculated and shown within each graph; letters indicate statistically significant differences. All raw data for the growth curves are presented in Data S2. (B) Proportion of cysts relative to total cells in *Colpoda* sp. cultures with and without 100 μM IAA treatment. (C) Cell size of *Colpoda* sp. with and without 100 μM IAA. (D) Total cell counts of *Colpoda* sp. and bacterial-depleted *Colpoda* sp., with and without 100 μM IAA. (E) Colony-forming unit (CFU) counts of bacteria in *Colpoda* sp. cultures with and without 100 μM IAA. (F) CFU counts normalized to protist number in *Colpoda* sp. cultures with and without 100 μM IAA. (G) CFU counts from the cell-free filtrate of *Colpoda* sp. cultures with and without 100 μM IAA. Box plots generated using six biological replicates per group. The center line indicates the median, the box represents the IQR, and the whiskers represent 1.5 × IQR. Asterisks denote statistical significance: ^*^P < .05, ^*^^*^P < .01, ^*^^*^^*^P < .001, as determined using a two-tailed Student’s t-test assuming equal variance.

Given the conservation of IAA production among the bacteria associated with our collection of diverse rhizosphere protist cultures, we hypothesized that the protists could have functional responses to externally sourced IAA. Supplementation with exogenous IAA in the absence of tryptophan increased the growth yield of all protists (Fig. 4A). Sensitivity to IAA differed among cultures. The opisthokont *Nuclearia* sp. and the amoeba *Flamella* sp. responded to the lowest tested dose (0.1 μM) and had the lowest maximal effect dose (1 μM). The three Stramenopile protists responded at 10 μM, whereas isolates from the supergroup Rhizaria responded only at 100 μM or greater. IAA’s effect size also varied among protists; the *Chrysophyte*, *Ochromonas* 1, *Ochromonas* 2, and *Poteriospumella* spp*.* from the Stramenopile lineage exhibited the greatest increase in cell density after IAA treatment (2.4–5.1-fold), whereas protists from other groups increased 1.2–2.2-fold in density. Two treatments of IAA, 0.001 and 0.01 μM, did not significantly differ from the control for any of the 10 protist species. Therefore, these data points were excluded from the figure (Fig. 4A), though full data are provided in Data S2. We also examined the growth rate of the protists. Exogenous IAA significantly reduced doubling times for *Thaumatomastix* and *Flamella* spp. across all dosages, indicating a robust response. In contrast, *Poteriospumella*, *Crysophyte*, and *Nuclearia* spp. growth rates were only influenced at high IAA concentrations, indicating a dose-dependent response in growth rate (Fig. 4A).

Having observed broad IAA-induced increases in growth yield but not consistently in growth rate, we hypothesized that IAA may be increasing the cell density at which protists transition from growth to cyst formation. Cysts, like bacterial spores, are a resting state for protist cells. Delaying encystment is a mechanism by which protists may reach a higher density, without altering their growth rate. We examined the timing of encystment in *Colpoda* sp., an isolate allowing clear morphological distinction of cysts from active cells. At 48 h after transfer, only 3% of cells in *Colpoda* sp. cultures treated with IAA had formed cysts, whereas untreated cultures were 42% encysted (Fig. 4B and S6).

IAA treatment also broadly increased the cell size of active protists, resulting in 35% greater imaged cell area of *Colpoda* sp. (Fig. 4C) and 12%–67% increases in six other protists (Fig. S7). Three species, *Chrysophyte* sp. (UC74), *Ochromonas* sp. 1 (UC81), and *Flamella* sp. (UC122), were excluded due to measurement limitations: UC74 and UC81 were too small to distinguish active cells from cysts, and UC122 displayed continuous shape changes that prevented consistent sizing. This finding indicates that IAA causes a delay in encystment and growth arrest of diverse protists, allowing the cells to reach increased population density in culture.

To investigate whether the protist response to IAA might be mediated by the bacteria in the cultures, e.g. by metabolizing IAA into a protist growth factor, we added IAA to the bacterial-depleted, IAA-nonproducing *Colpoda* sp. culture tested in Fig. 3B. Although protist cell counts were reduced in bacteria-depleted cultures, the IAA effect size was identical to nondepleted cultures, producing a 1.6-fold increase in protist cell density (Fig. 4D). This indicates that the response of *Colpoda* sp. to IAA is not dependent on an intact microbiome. We also asked whether IAA supplementation affects bacterial growth in the protist culture. IAA treatment of *Colpoda* resulted in an increase in the population of culturable bacteria in the *Colpoda* sp. culture, both per volume and per protist host (Fig. 4E and F). However, when bacterial cells were filtered out and separated from the *Colpoda* cells in culture, their population size was not affected by IAA supplementation (Fig. 4G). This further supports the involvement of the protist in the IAA response and suggests that IAA may provide a protist-dependent growth advantage to protist-associated bacteria.

### Synthetic auxins induce a response similar to indole-3-acetic acid

**Figure 5:**
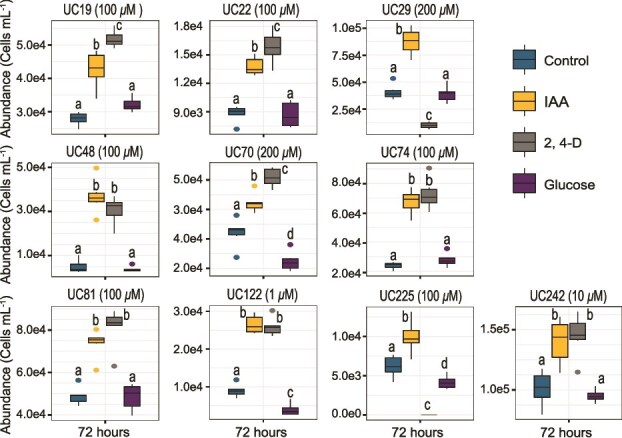
Influence of 2,4-D, and glucose on protist growth. Protist cultures were supplemented with either 2,4-D or glucose at concentrations matched to the IAA doses that positively influenced protist growth in Fig. 3A (as indicated in the panel title bars). Final cell numbers for each protist species were compared across IAA, 2,4-D, glucose, and untreated controls. Box plots generated using six biological replicates per group. The center line indicates the median, the box represents the IQR, and the whiskers represent 1.5 × IQR. Statistically significant differences among treatments are denoted by different letters (Tukey’s HSD post hoc test, α = 0.05).

To determine if the observed protist responses were specific to IAA or applied to other auxins, protist cultures were amended with 2,4-dichlorophenoxyacetic acid (2,4-D), a synthetic auxin commonly used as a broadleaf herbicide [65]. The 2,4-D treatment resulted in growth enhancement of nine protists, often increasing density to a greater degree than IAA (Fig. 5). The 2,4-D treatment was lethal to *Allapsa* sp., pointing to a potential taxon-specific sensitivity analogous to that of dicot plants. Addition of a common carbon source, glucose, did not increase the growth of any protist in the presence of heat-killed *E. coli* (Fig. 5), and *Colpoda* was unable to grow in the absence of heat-killed *E. coli* prey regardless of IAA supplementation (Fig. S8). This suggests that IAA-mediated growth phenotypes do not derive from any function of IAA as a labile carbon source. These collective observations support the hypothesis that IAA promotes protist growth and regulates encystment through a hormone-like mechanism. This response appears conserved across diverse lineages of heterotrophic protists, although taxon-specific variations exist. These findings suggest that auxin perception could be a common and conserved feature in many eukaryotic lineages.

### Candidate indole-3-acetic acid-response genes in protist genomes

**Figure 6:**
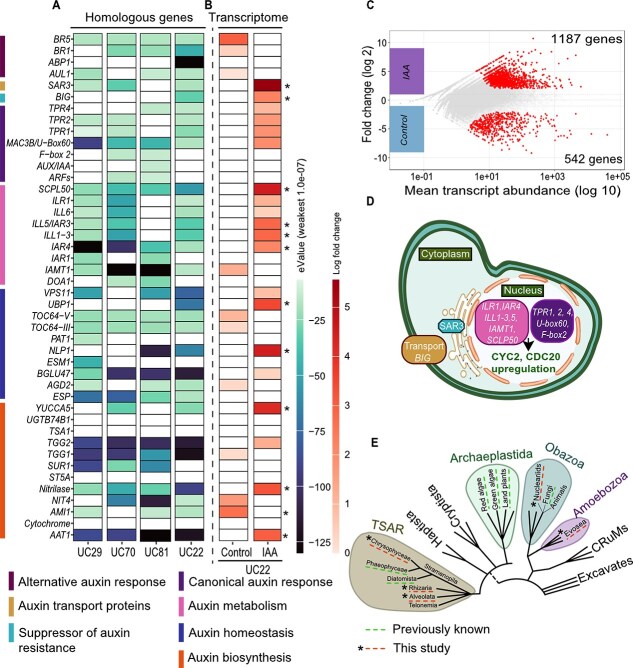
Presence of auxin-perception related genes in protists and their transcriptional response to IAA treatment. (A) Genes identified in protist sequences from *Ocheomonas* sp. 1, *Thaumatomastix* sp., *Ochromonas* sp. 2, and *Colpoda* sp. with homology to genes in auxin pathways. The presence of homologous genes was determined by local BLAST searches against *A. thaliana* reference sequences from the list of conserved auxin genes from [40, 41]. The shading of the boxes indicates the E-value of the best match in the genomes. (B) Transcript abundance of IAA-pathway genes in *Colpoda* sp. Box shading represents the log2-fold difference in transcript abundance between conditions, with significant differences in abundance indicated by asterisks. (C) MA plot showing the relationship between average normalized transcript abundance and log2 fold change between conditions for each transcript identified. Colored points indicate those transcripts with a significant difference in abundance due to IAA treatment. The number of transcripts enriched in each condition is indicated. A full list of differentially abundant transcripts and their putative identification is presented in Data S3. (D) This schematic diagram presents the aspects of the IAA-pathway genes and associated processes identified in protist genomes. To represent their potential localization within protists, we have projected plant cell-based functional localization onto a generalized protist cell. (E) Simplified eukaryotic tree highlighting groups. Dotted lines with stars mark previously known groups; dotted lines without stars denote new groups identified in this study.

The genetic basis of auxin response outside of photosynthetic organisms within the Archaeplastida is unknown. To determine whether heterotrophic protists harbor homologs of genes contributing to IAA perception, we assembled eukaryotic contigs from the metagenomes. Contigs representing estimated genome completeness of 50%–79% were recovered from four protist cultures, *Ochromonas* sp. 1 (77.25%), *Ochromonas* sp. 2 (74.51%), *Thaumatomastix* sp. (79.61%), and *Colpoda* sp. (52%). (Table S2). We searched the predicted genes from the contigs for similarity (≥30% translated nucleotide, identity over 85% query coverage, and E-value ≤1e^−7^) to a set of 44 *A. thaliana* genes with characterized roles in auxin metabolism, transport, resistance, and biosynthesis [40, 41] (Fig. 6A). The contigs from each protist culture encoded predicted genes with sequence homology to 27–33 of the auxin-related *A. thaliana* genes, of which 13 were present in all four protist assemblies. These included homologs to genes involved in auxin redox homeostasis (*IAR4*), processing and metabolism (*ILR1, IAMT*), and downstream gene regulation (*TPR2/4, MAC3B*) [66–69].

Given its robust growth response to supplemental IAA (Fig. 4A) and prevalence of predicted IAA pathway genes (Fig. 6A), we selected *Colpoda* sp. for an RNA-sequencing analysis to investigate transcriptional changes in response to IAA. This analysis identified 1729 differentially abundant transcripts (1.4% of the total predicted genes) in response to IAA exposure and the ensuing growth response, indicating a significant transcriptional remodeling (Fig. 6C). Only 16% of genes had predicted KEGG function, suggesting many functions of protist genes remain unresolved (Fig. S9). Among the transcripts identified as significantly different in abundance due to IAA treatment were 12 of the genes identified in *Colpoda* sp. as homologs to IAA pathway genes, 11 of which were elevated in abundance in the presence of IAA (Fig. 6B). The IAA pathway genes included candidate homologs to the auxin transport genes *BIG* and *SAR3,* and members of the ILL family of auxin homeostasis genes, *ILR1*, *IAR3/ILL5,* and *ILL1-3* (Fig. 6B) [66, 70–73]. Also among the differentially abundant transcripts were genes with homology to those involved in cell cycle progression, including *CYC2* and *CDC20* (Fig. 6D and Data S3), which mediate cell growth in response to auxin in plants and diatoms [74, 75]. Primarily, the results presented here support the notion that, in addition to the phenotypic observations of IAA-induced growth, several heterotrophic protists harbor conserved genetic signatures associated with IAA perception and response.

## Conclusion

In this study, we demonstrate that a diverse group of heterotrophic protists isolated from the maize rhizosphere are consistently associated with simple communities of bacteria that express multiple PGPR traits and synthesize the auxin hormone IAA. The high conservation of IAA production among the majority of recovered genomes, despite the relatively low overlap in the taxonomic composition of the communities, suggests a high degree of functional redundancy within these microbial assemblages. Functional redundancy is often seen as a sign that a particular trait is essential for the survival and persistence in that ecosystem [76]. Thus the conservation of IAA synthesis pathways in protist associated bacteria suggests this trait may play an important role in mediating bacterial-protist interactions. This finding significantly expands the scope of organisms exhibiting IAA-regulated growth. While previously recognized primarily in plants, auxin responses have been detected in several marine chlorophytes, diatoms, and plant associated fungi [74, 77–83] (Fig. 6E)*.* This study reveals that auxin perception and response also extends to single celled eukaryotes from diverse additional clades, including the Evosea clade (*Flamella* sp.) of Amoebozoa, the Nucleariid clade (*Nuclearia* sp.) of the Opisthokonts, the Rhizarians (*Cercomonas*, *Thaumatomastix*, and *Allapsa* spp.), and Ciliates (*Colpoda* sp.) (Fig. 6E). This expansion suggests an ancient evolutionary origin of IAA-regulated growth, likely predating the emergence of plants. Significantly, these results also extend the known environments for IAA-regulated growth beyond marine phytoplankton. We show that IAA-regulated growth is a common characteristic of bacterivorous heterotrophic protists isolated from the rhizosphere, which specifically associate with IAA-producing bacteria. As auxin-producing bacteria are hypothesized to utilize IAA to modulate plant health [64, 84], these findings place IAA as a central metabolite with the potential to regulate the tripartite interkingdom interactions between bacteria, plants, and protists in the rhizosphere.

## Supplementary Material

Supplementary_Figures_Tables_wraf234

Data_S1_wraf234

Data_S2_wraf234

Data_S3_wraf234

Supplementary_files_brief_summary_wraf234

Supplementary_Note_wraf234

## Data Availability

All raw sequencing data includes raw Illumina reads of metagenomes, raw Oxford Nanopore reads of 36 bacterial isolates for the whole-genome sequencing, and raw long-read data for the assembly of the *Colpoda* genome. Metagenome-assembled genomes, and bacterial whole-genome sequences generated in this study are publicly available through the NCBI BioProject database PRJNA1090919. Forty-eight MAGs have been submitted to NCBI under the same BioProject, and two additional MAGs with less than 90% completeness, which do not meet NCBI submission guidelines, are available upon request. The constructed contigs of *Colpoda* sp*.*, *Ochromonas* sp. 1, *Thaumatomastix* sp., and *Ochromonas* sp. 2 analyzed in this study are also available upon request. Raw Illumina reads from the transcriptome sequencing of *Colpoda* sp., with and without IAA treatment, have been deposited in the NCBI SRA under the same BioProject mentioned above. These are the KBase narratives links, Metgenome_10protists—KBase Narrative, used to generate contigs from raw Illumina reads and to assemble Bacterial MAG, qualitative assessments, and for taxonomic characterization. ANI_MAGs and Isolates_genome_quality__Identity_ANI were used to perform ANI analysis between the bacterial MAGs and bacterial isolates. UC22 and Euks_binning were used for protists genomes assembly. UC22_F_050724 was used for *Colpoda* sp. transcriptome analysis.

## Supplementary material

Supplementary information. Methods S1–S5, Figures S1–S9, Tables S1 and S2, and legends for Data S1–S3.
