# Supplementary material for: Diverse soil protists show auxin regulated growth in partnership with auxin-producing bacteria
Source: ISME J. 2025 Oct 16;19(1):wraf234. doi: 10.1093/ismejo/wraf234 (PMC12663960; doi:10.1093/ismejo/wraf234)
Supplement: Supplementary_Figures_Tables_wraf234 [file supplementary_figures_tables_wraf234.pdf]

Diverse soil protists show auxin regulated growth in partnership with auxin-producing bacteria

Ravikumar R. Patel, Lindsay R. Triplett, Stephen J. Taerum, Sara L. Nason, Cole O. Wilson, Blaire Steven

Blaire Steven

Email: Blaire.Steven@ct.gov

This PDF includes:

Figures S1 to S9

Table S1 and S2

Other supplementary materials for this manuscript include the following:

Data S1 to S3

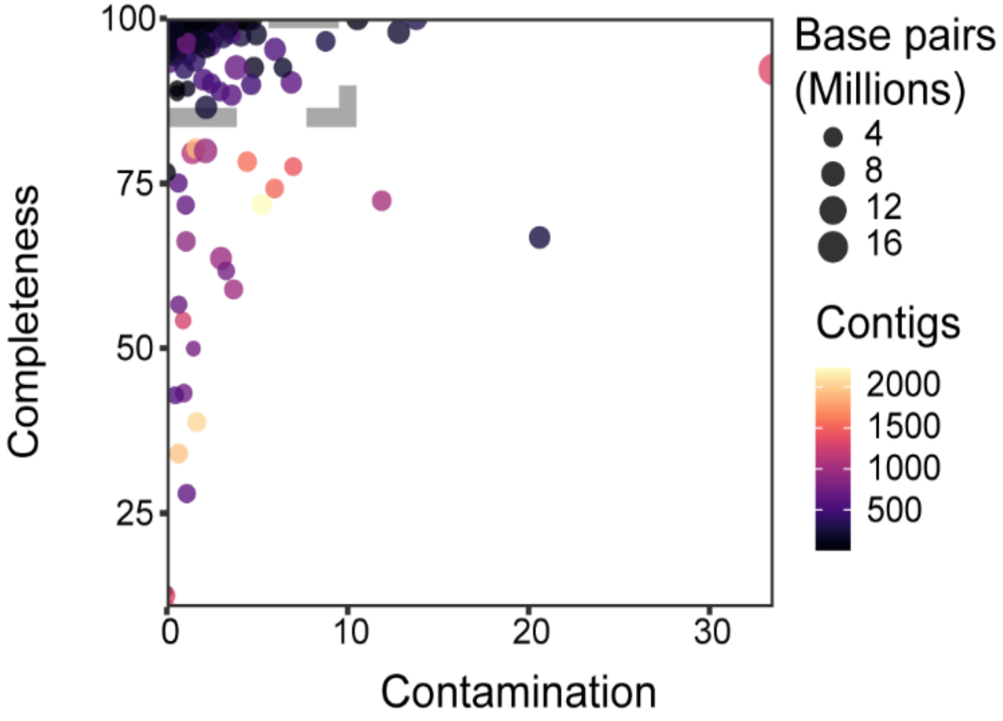

Figure S1. Quality of metagenome-assembled genomes. The dot plot represents completeness and contamination of MAGs, including merged MAGs. Each dot represents the individual genome, and the dots in the gray square represent the high-quality MAGs (> 85 % complete and < 10% contamination).

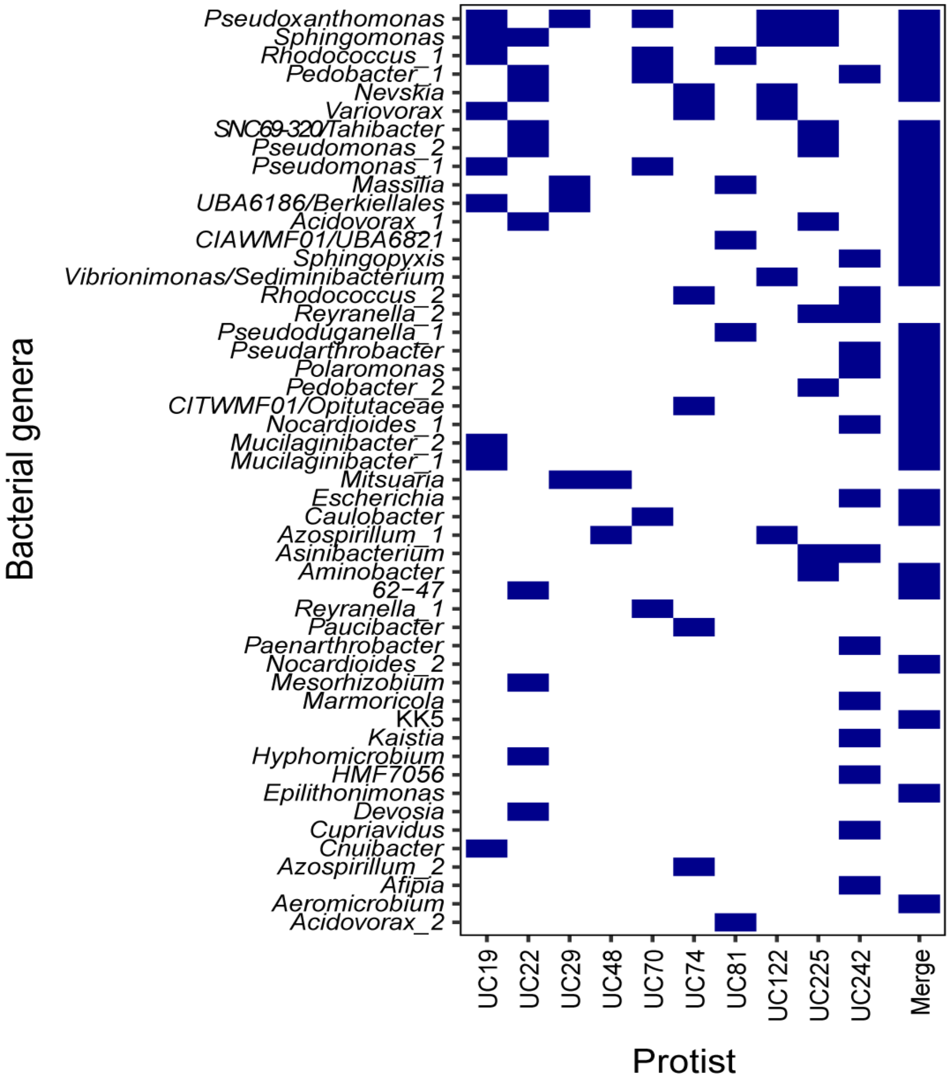

Figure S2. Presence-absence heatmap of MAGs assembled from protist culture metagenomes. Reads from each culture were assembled individually or as a combined dataset labeled ‘Merge’. A pairwise ANI analysis was performed to determine if MAGs were shared between datasets, and MAGs sharing > 95% nucleotide identity were considered the same.

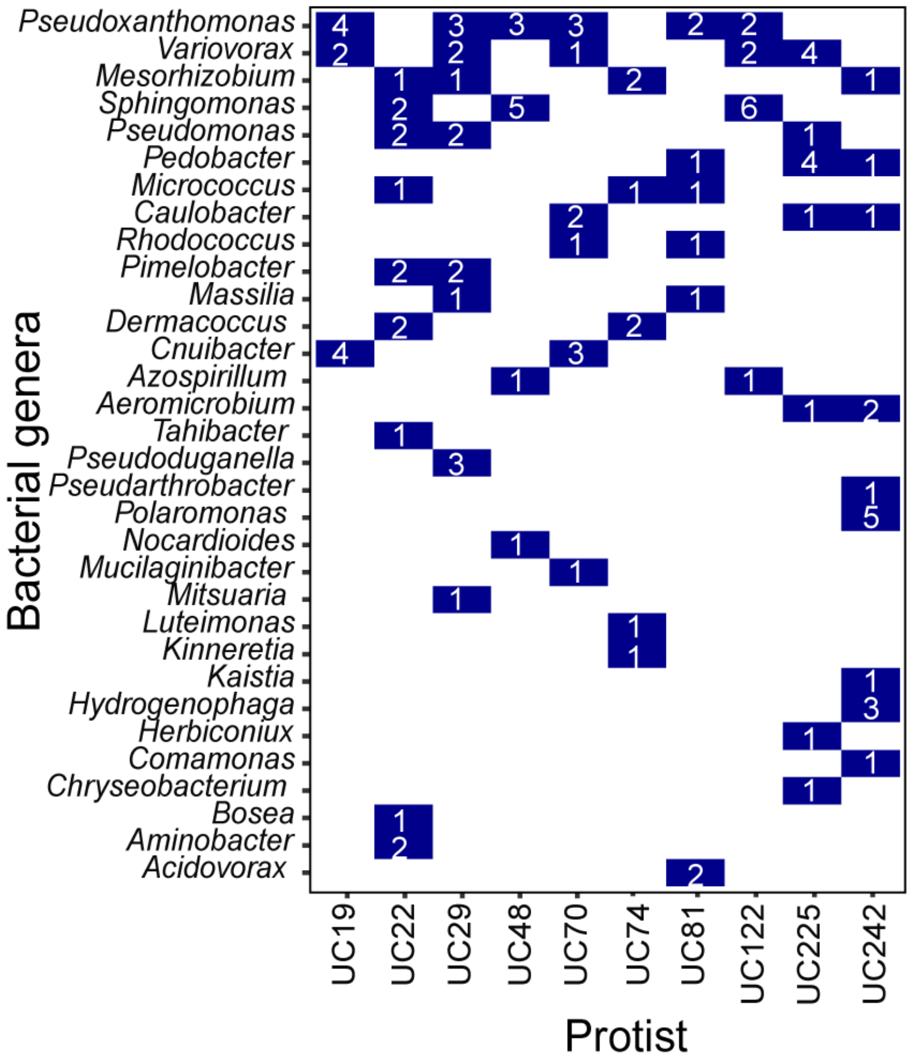

Figure S3. Presence-absence heatmap of bacterial isolates after identification using the 16S rRNA gene sequence. A total of 115 bacterial isolates were identified using 16S rRNA gene sequencing and classified to the genus level. Isolates belonging to the same genus were grouped and represented by a single entry for clarity in the figure.

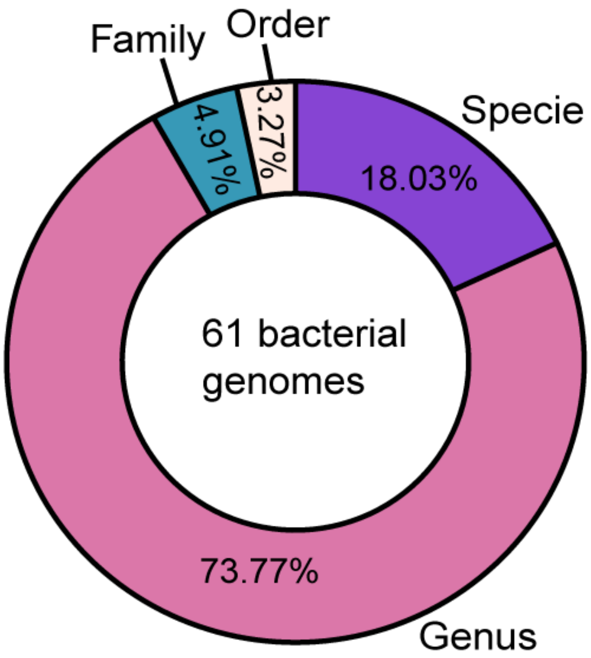

Figure S4. Taxonomic classification depth of protist-associated bacterial genomes. The pie chart summarizes the deepest taxonomic level to which each of the bacterial genomes was classified using the GTDB-Tk database. Each slice represents the proportion of genomes classified at a given taxonomic rank.

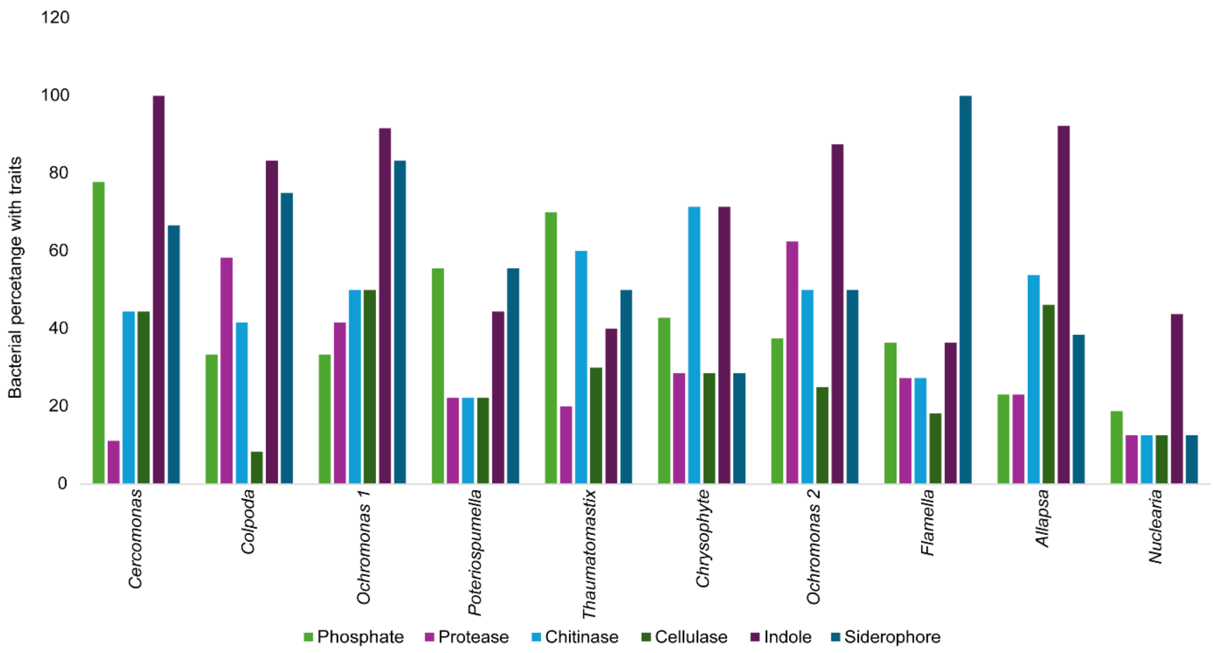

Figure S5. Qualitative analysis of PGP traits in 115 bacterial isolates associated with ten protist taxa. The percentage of bacterial genera positive for each PGP trait, phosphate solubilization, protease, chitinase, cellulase, indole production, and siderophore production, is shown for bacteria with individual protist hosts.

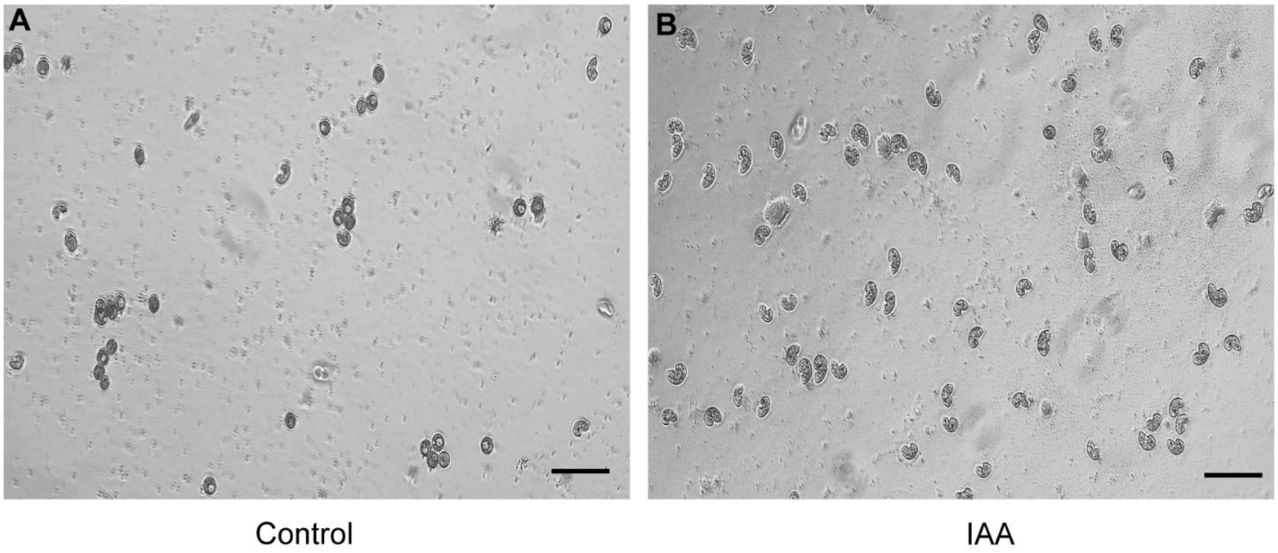

Figure S6. Bright-field microscopy images of *Colpoda* sp. with and without IAA. (A) Control; (B) Treated with 100 μM IAA after 48 hours of growth. Images were captured using a 10x objective. Scale bar = 100 μm.

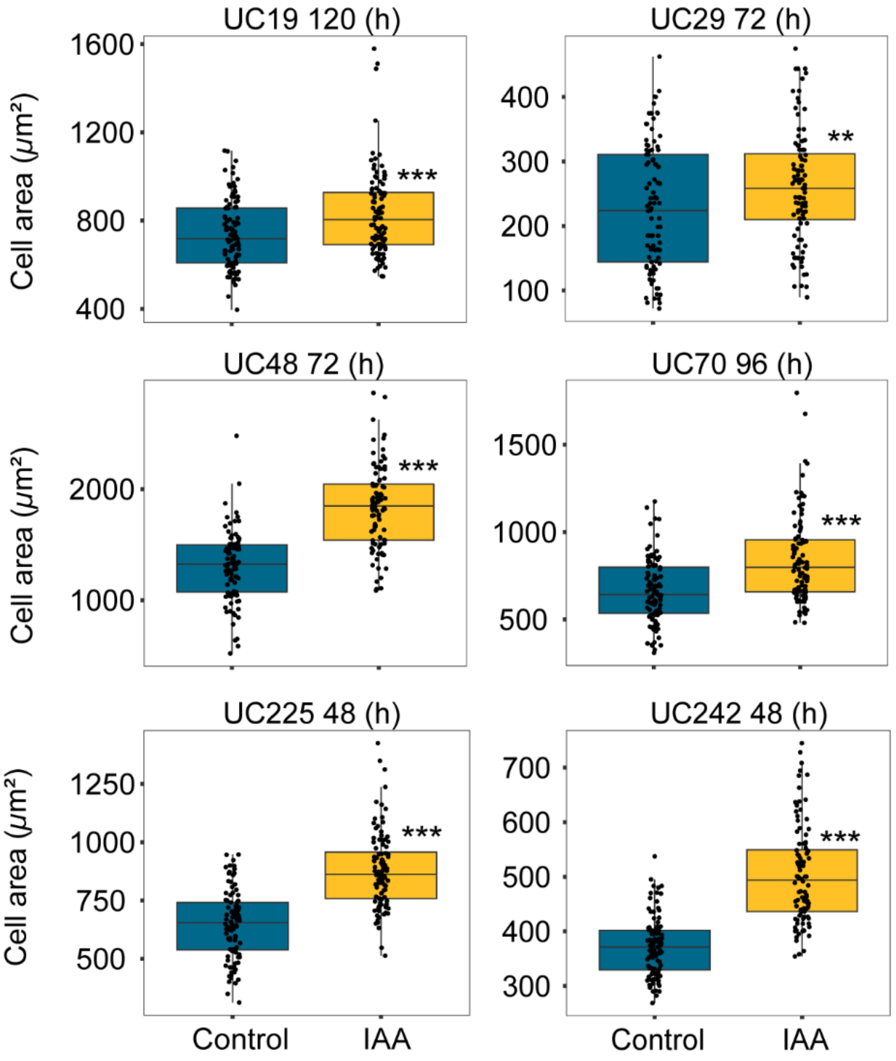

Figure S7. Effect of IAA on protist cell size. Cell size was measured for 100 randomly selected cells per treatment group (control and IAA-treated) using ImageJ software. Measurements were taken at time points corresponding to the active state of the majority of cells, as indicated in the graph. Asterisks indicate statistically significant differences based on two-tailed Student's *t*-tests assuming equal variance.

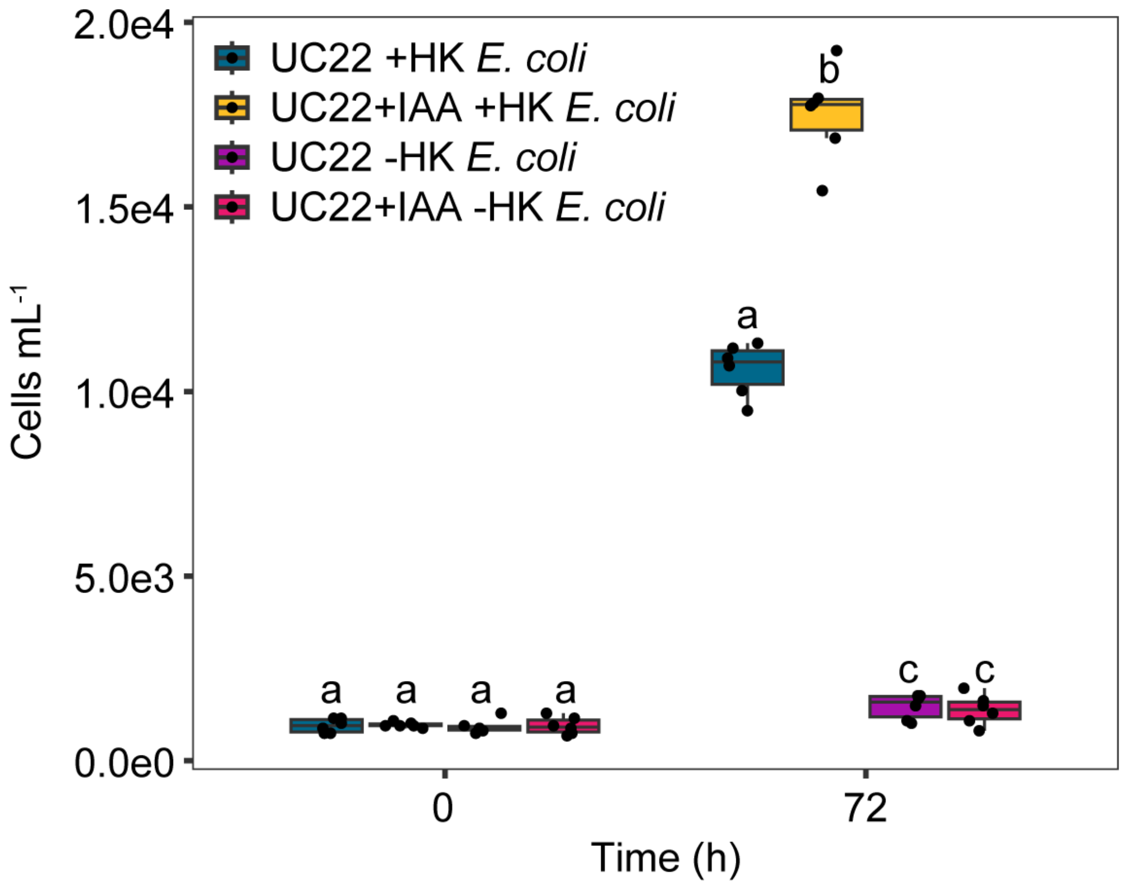

Figure S8. *Colpoda* sp. growth in response to heat-killed *E. coli* and IAA treatment. *Colpoda* sp. cells were incubated with or without heat-killed *E. coli* and exposed to 100 µM IAA at the start of the experiment. Cell counts were recorded at 0- and 72-hours post-treatment. The experiment was conducted with six biological replicates. Asterisks indicate statistically significant differences based on two-tailed Student's *t*-tests assuming equal variance.

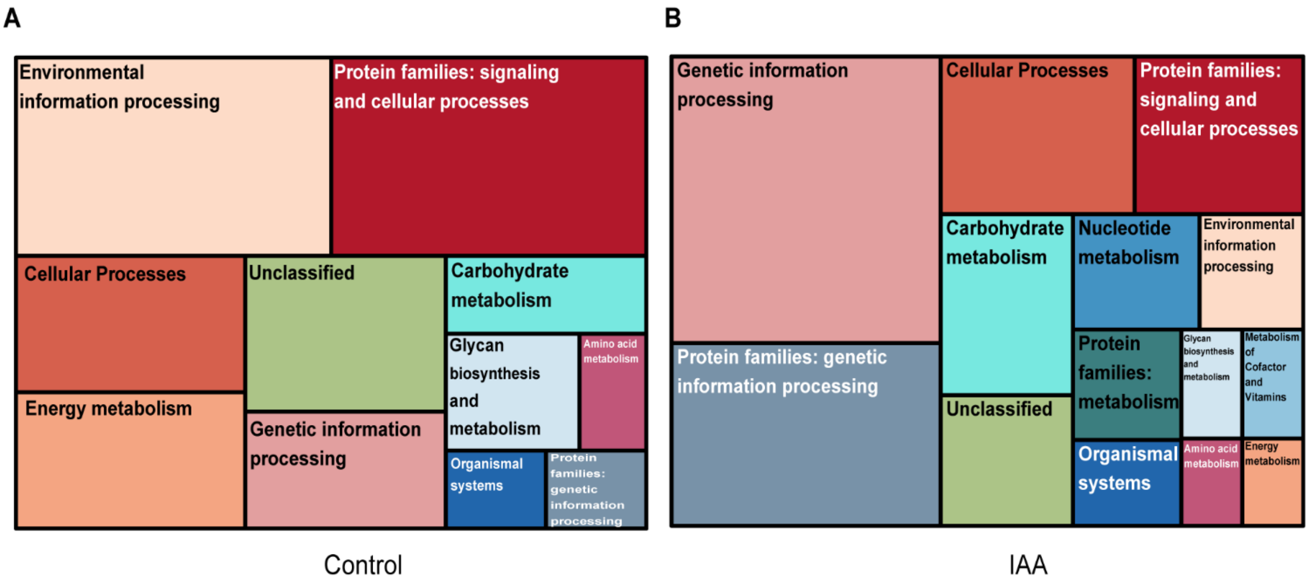

Figure S9. Treemap visualization of functional annotation of differentially abundant transcripts in *Colpoda* sp. using KEGG Ortholog. (A) Transcripts are significantly enriched in control conditions; (B) Transcripts are significantly enriched in IAA treatment. The size of each box represents the relative abundance of transcripts within each functional category.

Table S1. Classification of maize rhizosphere protists through 18S rRNA gene. The number of contigs generated from metagenome sequencing of eukaryotes, bacteria, and eukaryote organelles.

**Isolate name**	**Identity in NCBI 18S rRNA**	**Bacterial contigs**	**Eukaryote contigs**	**Organelle contigs**
UC19	*Cercomonas* sp. Strain SF75	3695	3341	1
UC22	*Colpoda* strain HCFF1288	7713	5701	35
UC29	*Ochromonas danica* strain CCMP588	1882	2196	6
UC48	*Poteriospumella lacustris*	840	85	5
UC70	*Thaumatomonas* sp. 6	749	1845	3
UC74	Chrysophyte	2112	3069	7
UC81	*Ochromonas sp.*	368	1500	5
UC122	*Flamella balnearia*	1789	3	3
UC225	*Allapsa vibrans* strain ATCC 50734	7118	3268	2
UC242	*Nuclearia leuckarti* strain LU21.6	2952	2905	313

Table S2. Four protist contigs, assembly quality, and statistics.

	***Colpoda* sp.**	***Ochromonas1* sp.**	***Ochromonas2* sp.**	***Thaumatomastix* sp.**
Genome size (bp)	63255866	18718969	23288013	27416207
Complete (BUSCO_v5)	36%	67.06%	65.49%	66.67%
Complete + Partial (BUSCO_v5)	52%	77.25%	74.51%	79.61%
GC content	34.99%	56.02%	54.28%	57.60%
Contig sequence number	6551	1487	997	1436
Maximum contig sequence length (bp)	193242	106632	190787	173395
Minimum contig sequence length (bp)	3000	3041	3229	3005
Average contig sequence length (bp)	9656	10035	23358	19092
Contigs N50 (bp)	14190	14872	32563	28234
