# Supplementary material for: Diverse soil protists show auxin regulated growth in partnership with auxin-producing bacteria
Source: ISME J. 2025 Oct 16;19(1):wraf234. doi: 10.1093/ismejo/wraf234 (PMC12663960; doi:10.1093/ismejo/wraf234)
Supplement: Supplementary_files_brief_summary_wraf234 [file supplementary_files_brief_summary_wraf234.docx]

**Supplementary Notes (PDF):** Additional experimental procedures and details supporting the main text, including, (Method S1) Protist morphology analysis, 18S and 16S rRNA primers and PCR procedure detail, (Method S2) IAA quantification by LC-HRMS, (Method S3) CFU enumeration protocol, and (Method S4) *Colpoda* sp. genome assembly pipeline, are provided in the Supplemental Methods to ensure clarity and reproducibility.

**Supplementary Figures and Tables (PDF):**

Combined supplementary file containing eight figures and two tables. Includes MAGs quality, bacterial and protist taxonomic profiles, IAA effects on *Colpoda* sp. and other protists, functional transcript annotation, and summary tables for ten protists’ taxonomy and genome statistics of four protists.

**Data S1.** ANI analysis between the MAGs, Final list of genomes with the taxonomic identification at the genus level, and ANI analysis between the MAGs and bacterial isolates. The cutoff ANI value is 95% or greater. Secretion systems Type I, II, III, IV, and VI genes annotation in 61 bacterial genomes.

**Data S2.** Raw cell number after treatment with seven different concentrations of IAA and control (without any treatment), and statistical analysis along with growth response with 2,4-D and glucose in comparison to control and IAA in all ten protists.

**Data S3**. List of genes up- and down-regulated in *Colpoda* sp. in response to IAA treatment. Differential expression analysis was performed using DESeq2; log2 fold change, p-values, and adjusted p-values (q-values) are provided along with their KEGG function annotation.
