# Supplementary material for: Diverse soil protists show auxin regulated growth in partnership with auxin-producing bacteria
Source: ISME J. 2025 Oct 16;19(1):wraf234. doi: 10.1093/ismejo/wraf234 (PMC12663960; doi:10.1093/ismejo/wraf234)
Supplement: Supplementary_Note_wraf234 [file supplementary_note_wraf234.pdf]

# Diverse soil protists show auxin regulated growth in partnership with auxin-producing bacteria

Ravikumar R. Patel, Lindsay R. Triplett, Stephen J. Taerum, Sara L. Nason, Cole O. Wilson, Blaire Steven

Blaire Steven

Email: Blaire.Steven@ct.gov

**This PDF includes:**

Method S1 to S5

**Method S1**

**Protists morphology analysis**

1 mL of protists was inoculated into 9 mL of fresh PAGE's buffer supplemented with 5.6 × $10^6$ heat-killed *E. coli* $mL^{-1}$ and cultured in sterile 50 mL polypropylene tubes (Thermo Fisher Scientific) for five days. For imaging, 500 µL aliquots were transferred to µ-Plate 24-well black plates with clear bottoms (ibidi USA). Morphology was observed using a Zeiss ID02 Invertoscope with an oil immersion lens, images captured with an Axiocam 305 color camera and processed in Zen 3.4 Blue Edition software.

**Method S2**

**18S rRNA Primers**

Protists UC19, UC22, UC29, UC70, UC74, UC122, UC225, and UC242 were amplified using the primer:

EukA: 5' AACCTGGTTGATCCTGCCAGT 3'

EukB: 5' TGATCCTTCTGCAGGTTCACCTAC 3'

Protist UC81 was amplified using the primer:

82F: 5' GAAACTGCGAATGGCTC 3'

1498R: 5' CACCTACGGAAACCTTGTTA 3'

**16S rRNA primer**

All the bacterial isolates' 16S rRNA genes were amplified using the primer:

27F (5′-AGAGTTTGATCCTGGCTCAG-3′)

1492R (5′-GGTTACCTTGTTACGACTT-3′)

**PCR reaction mixture and conditions for both 16S and 18S rRNA**

PCR reactions (50 µL) included 0.5 µL template DNA, 25 µL 2× Taq Master Mix (New England BioLabs, Ipswich, MA), 1 µL each of 10 µM primers, and nuclease-free water. Thermal cycling was performed with an initial denaturation at 94°C for 3 min; 30 cycles of 94°C for 45 s, touchdown annealing at 65°C decreasing 0.5°C per cycle for 30 s, and 72°C for 30 s; followed by a final extension at 72°C for 5 min. PCR products were confirmed on 0.8% agarose gels and

**Method S3**

***Colpoda* sp. long read sequencing and genome hybrid assembly**

The sequencing library was prepared using the Native Barcoding Kit 24 V14, and sequencing was conducted on a MinION flow cell (FLO-MIN14, R10 Version) with the Oxford Nanopore MinION MK1C platform. The genome was assembled using hybridSPAdes v3.15.376 [52] in the KBase, (UC22 genome assembly UC22-KBase_narrative). Genome completeness was assessed using BUSCO v5 in gVolante 61, yielding a 52% completeness.

**Method S4**

**Bacterial CFU count protocol**

CFU counts were assessed in the presence and absence of *Colpoda* sp., with and without IAA treatment. To prepare the *Colpoda* sp. filtrate, a 72-hour-old culture was passed through a 0.8 µm, 33 mm diameter, MCE, sterile, disposable membrane filter (MilliporeSigma, Burlington, MA, USA), generating a protist-free filtrate. This experiment consisted of four treatments: (1) *Colpoda* sp. (1000 cells $mL^{-1}$) in 2 mL of PAGE's, (2) *Colpoda* sp. (1000 cells $mL^{-1}$) in 2 mL of PAGE's with 100 µM IAA, (3) 1.7 mL of PAGE's supplemented with 300 µL of *Colpoda* sp. filtrate, which corresponds to the volume of *Colpoda* sp. culture required to achieve 1000 cells $mL^{-1}$ in first and second treatments and (4) 1.7 mL of PAGE's supplemented with 300 µL of filtrate and 100 µM IAA. In all treatments, $5.6 \times 10^7$ heat-killed *E. coli* was provided as a food source. After 48 and 72 hours of incubation, CFUs were enumerated on R2A agar plates. CFU counts in treatments containing protists were expressed as CFU $protist^{-1}$, whereas those in protist-free treatments were expressed as CFU $mL^{-1}$.

**Method S5**

**Ultra-high-performance liquid chromatography**

UltiMate 3000 ultra-high-performance liquid chromatography (UHPLC) system coupled to a Q-Exactive Orbitrap mass spectrometer (Thermo Fisher Scientific, Waltham, MA, USA) in positive electrospray ionization (ESI) mode.  Separation was achieved using an Agilent Zorbax 300 SB-C18 column (150 mm × 2.1 mm, 1.9 µm particle size with a mobile phase of 0.1% formic acid in water (A) and acetonitrile (B). A linear gradient from 6% to 95% B was applied between 3–11 min, followed by re-equilibration. Injection volume was 2 µL, with a flow rate of 200 µL/min, column temperature at 40°C, and autosampler at 10°C. IAA retention time was

8.46 min. The mass spectrometer was operated in Full MS/ddMS2 mode, with Full MS resolution at 70,000, scan range 120–500 m/z, AGC target of 3e6, and maximum IT of 50 ms.

Quantification used the [M+H]+ ion at 176.0706 m/z with TraceFinder version 4.1 EFS (Thermo Fisher Scientific). Calibration was performed with a seven-point standard curve (50–10,000 nM) using 1/x weighting. ISIS peak integration (area noise factor 5, peak noise factor 10) was applied, with manual curation. Mass accuracy was within 2 ppm, and retention time deviation was within 0.03 min of standards.
